# Professor Thomas Börner: the pioneer of molecular plant genetics in East Germany turns 80!

**DOI:** 10.1007/s00438-026-02498-w

**Published:** 2026-08-25

**Authors:** Christian Schmitz-Linneweber, Reimo Zoschke

**Affiliations:** 1 Molecular Genetics, Humboldt-University of Berlin, Philippstrasse 13, 10115 Berlin, Germany; 2 Max Planck Institute of Molecular Plant Physiology, Am Mühlenberg 1, 14476 Potsdam-Golm, Germany; 3 Plant Physiology, Humboldt-University of Berlin, Philippstrasse 13, 10115 Berlin, Germany

The 80th birthday of Professor Thomas Börner provides an opportunity to celebrate one of the architects of modern plant molecular genetics in Germany (Fig. 1). Over more than five decades, his pioneering discoveries in organelle genetics, his leadership during a transformative period in German science, and his dedication to mentoring generations of researchers have left an enduring impact on plant biology. For those who have worked with him, he is not only an outstanding scientist but also a generous mentor whose influence continues through the many scientists he has inspired.

**Fig. 1:**
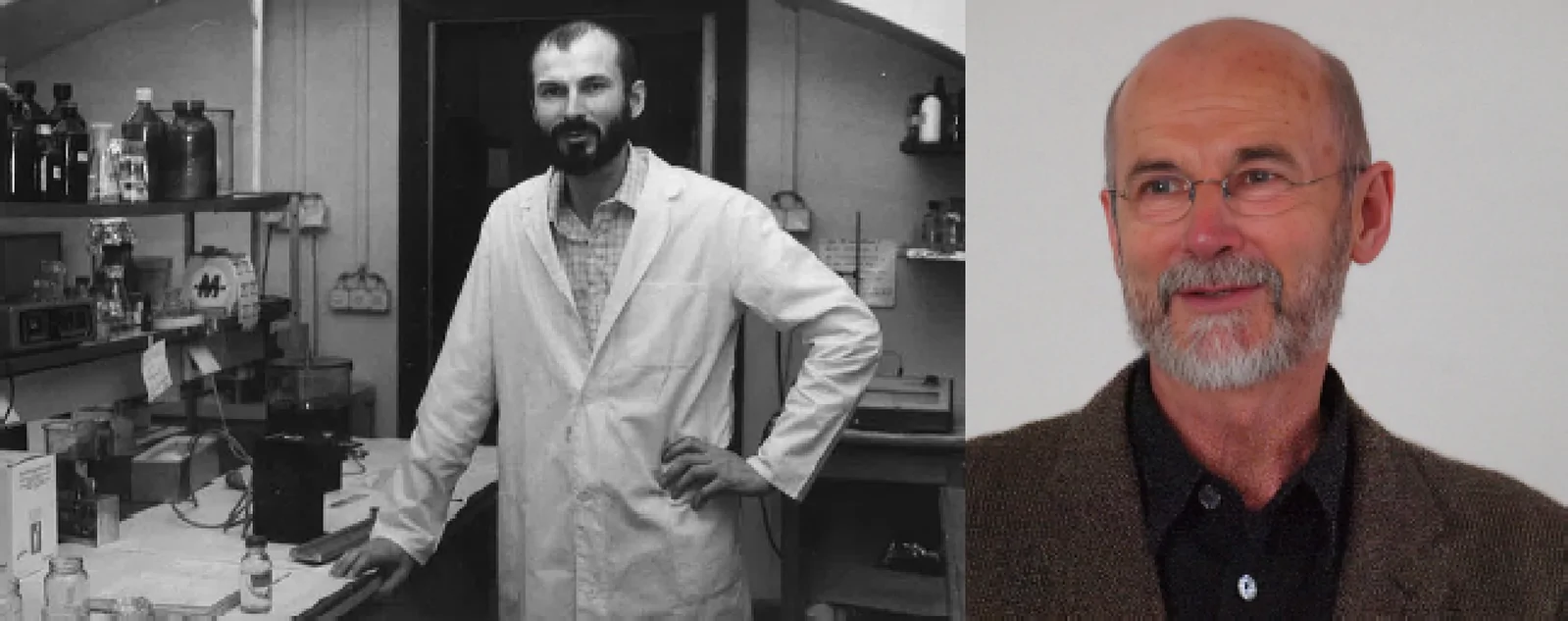
Professor Thomas Börner in his cellar laboratory in East Berlin in the 1980s and today

Born on July 11, 1946, in Leipzig, Tom Börner studied biology from 1965 to 1969 at Martin Luther University Halle-Wittenberg, where he joined the renowned group of Prof. Rudolf Hagemann. There he encountered one of the most fascinating questions in biology: how genetic information is organized and expressed beyond the nucleus, a question that would accompany him throughout his scientific life. In 1974, he completed his doctoral work at Martin Luther University Halle-Wittenberg on the effects of mutations in the chloroplast genome of higher plants. His habilitation thesis (1979) focused on the interaction between plastid genes and the nucleus during chloroplast biogenesis. In this period, he discovered that signals emerging from chloroplasts control the expression of nuclear genes that encode chloroplast proteins, one of the earliest demonstrations of what is now recognized as plastid-to-nucleus retrograde signaling (Börner 2017). Highly unusual for that time in East Germany, in 1977, Tom received the permission to travel to the UK to visit labs and conduct research on plant organelles. His findings were published in *Nature* (Bradbeer, Atkinson et al. 1979), making Tom Börner one of the few scientists behind the Iron Curtain to publish in such a leading journal of the Western scientific establishment.

In 1982, he moved to Humboldt-University of Berlin, where he was appointed as a full-time lecturer in genetics and head of the newly established Genetics Division, which he built up in the following years within the Biology Section. In 1984, he was appointed as a full professor of Genetics in Berlin. During this time, he established and led a thriving center for molecular genetics research, shaping the University’s profile in modern molecular biology in an academic environment that was soon to change dramatically. Even before the fall of the Berlin Wall, he established a lively academic exchange with colleagues from West Germany and West Berlin, particularly at the newly founded “Institut für Genbiologische Forschung GmbH” (Institute for Gene-Biology Research) headed by Prof. Lothar Willmitzer and Prof. Axel Brennicke, who conducted research in plant molecular genetics on the other side of the wall. The fall of the Berlin Wall on November 9, 1989, gave this long-standing scientific openness an entirely new historical significance. At the same time, however, the political transformation that followed was deeply disruptive: after German reunification, the employment contracts of all professors at Humboldt-University were terminated without exception. Owing to his outstanding scientific achievements and his independent, principled character, Tom Börner was among the few to secure a professorship, again through a highly competitive selection process. As part of this procedure, he was required to present his laboratory and facilities to the very candidates competing to replace him — a paradoxical situation that seems to have been more evident to him than to the selection committee, and that vividly illustrates the extraordinary circumstances surrounding German reunification. During these years, he became one of the key figures shaping the renewal of plant genetics and molecular biology in the Berlin metropolitan area. In a visionary approach, he convinced the DFG in 1999 to fund the SFB 429, a major collaborative research center entitled “Molecular Physiology, Energetics, and Regulation of Primary Metabolism in Plants”. This initiative, for the first time, brought together research in molecular plant biology at the three major universities of Berlin (Humboldt-University, Free University and Technical University), institutions that had previously been separated by the Berlin Wall, as well as at the newly founded University of Potsdam and the newly established Max Planck Institute of Molecular Plant Physiology in Potsdam-Golm. Tom Börner acted as spokesperson for the SFB 429 from 1999 to 2010.

Tom Börner’s success is particularly impressive given the scarcity of resources in East Germany at a time of severe economic constraints. Due to limited supplies, it was not uncommon to wash disposable gloves or reaction tubes and tips for reuse. His first laboratory was located in the ecology department in a rear building on Hermann-Matern-Straße (today Luisenstraße), until the department moved to the basement of a side wing of the Museum of Natural History on Invalidenstraße, a location that was regularly flooded during heavy rains. This problem was resolved when the Börner lab moved to the first floor, and conditions improved further when Tom and his group relocated to modern molecular biology laboratories on Chausseestraße in the 1990s. Despite these practical “inconveniences” and political constraints, he laid the foundation for a remarkable scientific career.

Tom Börner’s early work made foundational contributions to the understanding of chloroplast genetics, particularly the structure, expression, and inheritance of plastid DNA and its interaction with the nuclear genome (e.g., Börner et al. 1972; Börner et al. 1973; Herrmann et al. 1980; Siemenroth et al. 1980; Metzlaff et al. 1981; Siemenroth et al. 1981; Dorne et al. 1982; Metzlaf et al. 1982; Börner et al. 1986). These studies helped to establish key concepts of plastid molecular genetics in higher plants. He further advanced the field through investigations of plastid transcription and RNA metabolism, elucidating how gene expression in chloroplasts is coordinated with nuclear control mechanisms (e.g., Hess et al. 1993; Hess et al. 1994; Hess et al. 1994; Hübschmann et al. 1996; Vogel et al. 1997; Vogel et al. 1997; Hübschmann and Börner 1998; Vogel and Börner 2002; Zhelyazkova et al. 2012). In addition, Thomas Börner and his research group substantially advanced our understanding of plant mitochondria by uncovering key aspects of mitochondrial genome organization, replication, transcription, and organelle-nucleus interactions; their studies thereby provided fundamental insights into how mitochondrial genes are expressed and coordinated with nuclear genetic programs in plants (e.g., Backert et al. 1996; Backert et al. 1997; Backert et al. 1997; Backert and Börner 2000; Lisowsky et al. 2002; Emanuel et al. 2004; Emanuel et al. 2006; Preuten et al. 2010). A major achievement of his group in the plant organelle field was the identification and functional characterization of nucleus-encoded plastid and mitochondrial RNA polymerases, providing important insights into the dual genetic control of plant organellar gene expression (e.g., Hedtke et al. 1997; Weihe et al. 1997; Hess and Börner 1999; Hedtke et al. 2000; Hedtke et al. 2002; Lisowsky et al. 2002; Richter et al. 2002; Emanuel et al. 2004; Liere et al. 2004; Emanuel et al. 2006; Kühn et al. 2007; Kühn et al. 2009; Yin et al. 2009; Zhelyazkova et al. 2012; Börner et al. 2015). In a review in 2017, Tom described in detail how he started to study barley albino mutants in 1972 and found, among others, the variegated “*albostrians*” mutant lacking plastid ribosomes (Börner 2017). This mutant was the topic of numerous publications, while the underlying mutation remained enigmatic for almost half a century (Börner 2017). After decades of genetic research by Tom and his co-workers, the mutated gene could be identified only in 2019, seven years after Tom’s official retirement, as a homolog of the *Arabidopsis* CONSTANS, CO-like, and TOC1 (CCT) Motif transcription factor gene CHLOROPLAST IMPORT APPARATUS2 (Li et al. 2019).

Extending beyond plant organelles, Tom broadened his research to cyanobacteria, where his group contributed to the discovery and characterization of phytochrome-like photoreceptors, demonstrating their evolutionary conservation and functional relevance in prokaryotes (Hughes et al. 1997; Lamparter et al. 1997). In parallel, his pioneering work on non-ribosomal peptide synthesis in cyanobacteria linked secondary metabolism to ecological and evolutionary processes (e.g., Meissner et al. 1996; Dittmann et al. 1997; Neilan et al. 1999; Rohrlack et al. 1999; Kaebernick et al. 2000; Tillett et al. 2000; Christiansen et al. 2001; Dittmann et al. 2001; Dittmann et al. 2001; Sielaff et al. 2003; Dittmann and Börner 2005).

Even beyond this already extended research profile, Tom also ventured into microbiology, especially mycology, where he built up a team of researchers who were among the first to apply genetic typing to microorganisms, developing DNA fingerprinting for the differentiation, identification, and taxonomy of filamentous fungi and yeasts (e.g., Meyer et al. 1991; Lieckfeldt et al. 1992; Meyer et al. 1992; Meyer et al. 1992; Lieckfeldt et al. 1993; Meyer et al. 1993; Schlick et al. 1994; Kuhls et al. 1995; Kubicek et al. 1996; Kuhls et al. 1996; Kuhls et al. 1997; Kuhls et al. 1999).

Together, his contributions bridged plant genetics and molecular biology, microbiology, and ecological-evolutionary research, highlighting Tom’s scientific breadth and his diverse interests across and beyond the green lineage.

Alongside his research, he assumed numerous leadership roles and functions in academia. Tom headed the Interdisciplinary Center for Biotechnology at Humboldt-University (1985–1991), served as dean of the Department of Biology (during the pivotal years 1989–1991), and acted as spokesperson for the SFB 429 mentioned above (1999–2010). In addition, he served as a member of the Biotechnology Working Group of the East German Research Council (1988–1990), as a board member of the Commission for Research on In-Vitro-Recombination of Genetic Material (1988–1990), as a member of the Scientific Advisory Board of the Federal Institute of Plant Genetics and Crop Plant Research (1993–1998), as vice president and member of the executive board of the Society for Genetics (1991–1999), and as a review board member for the Alexander von Humboldt Foundation (1994–2004) and the DFG’s Botany Division (2004–2012). Recognition of his scientific stature came through numerous honors, including prestigious awards (e.g., Carl Correns medal 1980, Goethe-Preis 1988, Miescher-Ishida Prize 2010) and the election to leading academies such as the Berlin-Brandenburg Academy of Sciences and the German National Academy of Sciences Leopoldina.

While Tom’s scientific and academic achievements are formidable, his legacy is equally defined by his exceptional commitment to mentorship. Over decades, he trained and guided a large number of students and young scientists, many of whom have gone on to become professors and leaders in academia and research institutions themselves (see the list of alumni who sent birthday wishes below). His mentoring style combined intellectual rigor with genuine personal support: he encouraged independence, critical thinking, and scientific curiosity. Tom fostered an environment that gave students and mentees the freedom to shape their own scientific paths while benefiting from his outstanding expertise. This confidence in his scholars was coupled with an exceptional integrity, enduring and infectious passion for science as well as an openness to new ideas which continue to inspire those around him. Rather than leading through pressure, he offered calm, thoughtful advice, and met students and peers alike with the same respect and sense of equality. His dry and thoughtful humor often put setbacks into perspective, reminding his students that, in the end, it was only science — and that a failed experiment was simply part of the process, not a cause for discouragement. Importantly, Tom’s mentorship extended beyond formal supervision. He actively supported careers, built networks, and remained a trusted advisor long after his students had established their own laboratories. Even after his retirement in 2012, Thomas Börner has remained an active and respected presence in the scientific community. At the age of 80, Thomas Börner is a significant figure in the development of molecular plant genetics in Germany, whose influence continues through a vibrant scientific lineage shaped by the many researchers he has guided. Celebrating Professor Börner’s 80th birthday means honoring not only his scientific achievements, but a distinctive way of doing science — one that continues to influence his field and the people working in it.

**Happy Birthday Tom from your scholars and friends!**

Prof. Ilka Axmann (Heinrich Heine University Düsseldorf, Germany).

Prof. Steffen Backert (University of Erlangen-Nuremberg, Germany).

Dr. Petya Berger, née Zhelyazkova (University of Münster, Germany).

Dr. Alexandra-Viola Bohne.

Dr. Liliana Borsellino.

Dr. Guntram Christiansen (Miti Biosystems, Vienna, Austria).

Dr. Yuri Churin (University of Giessen, Germany).

Dr. Emilia Cincu.

Dr. Heide-Marie Daniel (BCCM/MUCL Agro-food & Environmental Fungal Collection, UCLouvain, Louvain-la-Neuve, Belgium).

Prof. Elke Dittmann (University of Potsdam, Germany).

Dr. Dan Enke (Simris Biologics GmbH, Berlin, Germany).

Dr. Heike Enke (Simris Biologics GmbH, Berlin, Germany).

Dipl.-Biochem. (digitalis) Christian Griebel (Technical University of Darmstadt, Germany).

Dr. Boris Hedtke (Humboldt-University of Berlin, Germany).

Prof. Wolfgang R. Hess (University of Freiburg, Germany).

Dr. Thomas Hübschmann.

Dr. Janina Krueger, née Apitz.

Dr. Katrin Kuhls.

Prof. Kristina Kühn (Martin Luther University Halle-Wittenberg, Germany).

Dipl.-Biol. Dietmar Lieckfeldt.

Dr. Karsten Liere (amedes genetics, Berlin, Germany).

Dr. Vanessa Loiacono (Charles University Prague, Czech Republic).

Mario Lorenz (Charité Berlin, Germany).

Dr. Martin Meixner.

Prof. Wieland Meyer (Westerdijk Fungal Biodiversity Institute KNAW, Utrecht, Netherlands).

Prof. Michaela Müller-McNicoll (Goethe University Frankfurt, Germany).

Dr. Tobias Preuten.

Dr. Uwe Richter (Newcastle University, United Kingdom).

Dr. Gabriele Saretzki (Newcastle University, United Kingdom).

Prof. Christian Schmitz-Linneweber (Humboldt-University of Berlin, Germany).

Dr. Johanna Sobanski (ICON plc, Berlin, Germany).

Prof. Jörg Vogel (Helmholtz Institute for RNA-based Infection Research, Würzburg, Germany).

Dr. Andreas Weihe.

Prof. Annegret Wilde (University of Freiburg, Germany).

Dr. Maria Yamburenko.

Dr. Reimo Zoschke (Max Planck Institute of Molecular Plant Physiology, Potsdam-Golm, Germany).

Dr. Yan Zubo (Goeppert LLC, USA).

## Data Availability

No datasets were generated or analysed during the current study.
